# The Emerging Role of Urease as a General Microbial Virulence Factor

**DOI:** 10.1371/journal.ppat.1004062

**Published:** 2014-05-15

**Authors:** Julian C. Rutherford

**Affiliations:** Institute for Cell and Molecular Biosciences, Medical School, Newcastle University, Newcastle upon Tyne, United Kingdom; Duke University Medical Center, United States of America

Urea is generated in humans following the breakdown of amino acids and is evenly distributed throughout the body, including in the central nervous system, subcutaneous adipose tissue, blood serum, and epithelial lining fluid [1], [2]. Various pathogenic microbes are able to utilise urea as a nitrogen source through the activity of the enzyme urease that converts urea into ammonia and carbamic acid, with the spontaneous hydrolysis of carbamic acid to carbonic acid generating a further ammonia molecule.

CH_4_N_2_O+H_2_O→NH_3_+CH_3_NO_2_

CH_3_NO_2_+H_2_O→NH_3_+H_2_CO_3_

Under physiological conditions the proton of carbonic acid dissociates, and the ammonia molecules become protonated to form ammonium, causing an increase in local pH that can interfere with host function. The role of urease in the virulence of some bacterial pathogens is well established; however, more recent studies are beginning to highlight the function of urease during human fungal infections, suggesting that this enzyme has a wide role during microbial infection.

## Established Roles for Urease during Microbial Infection

Urease activity is the basis of acid acclimation that enables *Helicobacter pylori* to colonise the acidic environment of the human stomach. In response to the acidification of the periplasm of *H*. *pylori*, a proton gated urea channel (UreI) imports urea into the bacterial cytosol, where it is hydrolysed by urease [3]. Ammonia and bicarbonate (following the conversion of carbonic acid to bicarbonate) are then utilised to buffer protons within the periplasm [4]. Without this mechanism, *H. pylori* is unable to establish infection in the stomach [5], [6]. The urease-derived ammonia is also toxic to host epithelial cells, resulting in cell damage at the sites of *H. pylori* infection [7].

A distinct urease-dependent process is associated with bacterial urinary tract infections, including those caused by *Proteus* and *Klebsiella* species. Infection by these urease-positive bacteria can result in the development of infection stones that surround and protect the pathogen. Stones form due to the precipitation of the minerals struvite and carbonate apatite, which are produced by the binding of ammonium to magnesium ions and bicarbonate to calcium ions, respectively [8], [9]. As with *H. pylori* infection in the stomach, ammonia has an additional role in urinary tract infections by causing damage to the glycosaminoglycan surface of the urothelium that protects epithelial cells from bacterial infection [10], [11]. Therefore, urease-mediated pH changes and damage to host epithelial cells are associated with the promotion of some bacterial infections.

## Emerging Pathogenic Roles for Urease during Fungal Infections

Studies of two evolutionarily diverse fungi, *Cryptococcus neoformans* (a basidiomycete) and *Coccidioides posadasii* (an ascomycete), suggest that urease has conserved roles in promoting bacterial and fungal infections. The inhalation and subsequent germination of fungal spores is the route of entry into the body for many fungal pathogens. Successful infection of the lung requires the pathogen to evade the host immune system, and subsequent dissemination is dependent on the ability of the fungus to move from the lungs to other organs via the bloodstream. *C. neoformans* and *Co. posadasii* are saprophytic yeast that infect humans via the lungs and cause disease, with the severity of infection correlating with a loss of host immune function, and in both cases, the disseminated form of infection is potentially life threatening [12], [13]. Studies of both *C. neoformans* and *Co. posadasii* suggest that urease-dependent pH changes are involved in immune system evasion and that ammonia toxicity to host cells promotes systemic disease.

Urease is required for the full virulence of *C. neoformans* and *Co. posadasii* in animal models of disease [14], [15]. Urease-positive strains of both yeasts promote host responses that are consistent with a nonprotective Type 2 (Th2) rather than a fungicidal Type 1 (Th1) immune response [15], [16]. Consequently, mice infected with urease-positive *C. neoformans* have higher levels of serum IgE, Th2 cytokines, and alternatively activated macrophages as compared to control mice infected with a urease-negative strain [16]. Furthermore, a Th2 host response may also provide the pathogen with additional host-derived urea as alternatively activated macrophages convert arginine to ornithine and urea [17]. The detection of urease-dependent increases in arginase expression and higher levels of urea at sites of *Co. posadasii* infection support this positive feedback model [15], [16].

One mechanism to account for the urease-dependent Th2 polarisation of the host immune system is that the pH changes associated with urease activity cause a reduction in the acidification and maturation of phagolysosomes in phagocytic cells, resulting in a loss of pathogen killing and antigen presentation. A range of studies are consistent with this model. Fungi excrete ammonia as part of their internal pH control, and localised increases in pH are associated with *Co. posadasii* infection [15]. Ammonia prevents phagosome and lysosomal fusion and maturation in mouse peritoneal macrophages [18]. Immature dendritic cells promote Th2 polarisation, and higher levels of this class of antigen-presenting cells are found in mice infected with urease-positive rather than urease-negative *C. neoformans* [16]. Consistent with these findings are bacterial studies of urease-deficient *Mycobacterium bovis*, which localise more efficiently with lysosomes than a parental wild-type strain [19]. Furthermore, *M. bovis*-derived ammonia accumulates in macrophages and correlates with the reduction in the cell surface trafficking of the major histocompatibility complex class II [20].

The dissemination of a fungal pathogen from the lungs to other organs will be facilitated by damage to epithelial cells that allow pathogen access to and from the bloodstream. Ammonia is toxic to mammalian cells, and the urease-dependent damage to human epithelial cells by bacterial pathogens suggests that the ammonia produced by urease-positive fungi may also promote fungal dispersal [7], [10], [11], [21]. Evidence to support this comes from two studies involving *C. neoformans*. Urease activity negatively influences the integrity of endothelial junctions in vitro, as assayed using human brain microvascular endothelial cells [22]. Urease also promotes the crossing of *C. neoformans* across the blood-brain barrier in vivo after the pathogen becomes trapped in the small capillaries of the mouse brain [23]. Therefore, a plausible model is that circulating host urea is metabolised by yeast trapped in the small capillaries of the brain, resulting in the export of ammonia. The localised death of host epithelium cells then results in a loss of integrity of the blood-brain barrier, allowing *C. neoformans* to migrate into the brain parenchyma. A prediction is that pathogen-derived ammonia has the potential to kill host cells throughout the body.

**Figure 1:**
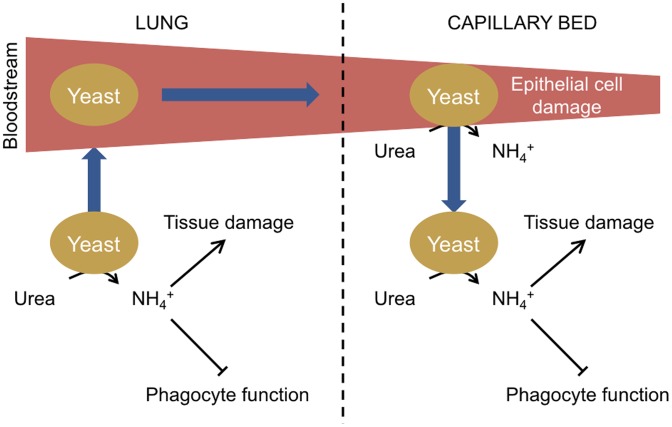
Model of urease function during fungal infection via the lungs. Following the inhalation of spores, yeasts germinate in the lungs. If the initial infection is not cleared due to a defective host immune response, yeast cells proliferate. Urea is present in the epithelial lining fluid of the lungs, and the urease-dependent secretion of ammonia from yeast cells further inhibits immune function by impairing phagocyte function and contributes to lung tissue damage. Individual yeast cells gain access to the bloodstream in damaged areas and circulate in the body until becoming trapped in small capillary beds. Circulating urea is then metabolised by the trapped yeast, resulting in ammonia secretion and damage to the epithelial cells of the capillary beds, enabling yeast cells to cross and proliferate in host organs.

The studies relating to the ammonia repression of phagocyte function and localised tissue damage suggest a general model for the role of urease during fungal infections (Figure 1). Initially inhaled fungal spores germinate and fungal cells are able to proliferate in imunocompromised individuals due to a reduction in host immune function. Metabolism of host-derived urea results in the excretion of ammonia from fungal cells, which contributes to host tissue damage and further inhibition of the host immune system by repressing phagocyte function. Fungal cell access to the bloodstream is facilitated by damage to lung tissue allowing distribution of fungal cells throughout the body, which become trapped in small capillary beds. Urease-dependent ammonia secretion then causes damage to capillary epithelial cells, enabling fungal cells to traverse from the bloodstream into organs where continued urease activity promotes infection.

## Urease as a Therapeutic Target

Urease was the first protein to be identified as a nickel enzyme [24]. Humans do not contain urease, and no human nickel enzymes are known, making urease a potential therapeutic target. Notably, the maturation of urease is a complex process involving a number of accessory proteins that are involved in the delivery and insertion of nickel into the active site of the enzyme, which contains two nickel atoms bridged by a carbamylated lysine residue. An impressive range of in vivo and in vitro studies of urease maturation in different bacteria, including *H. pylori*, *Sporosarcina pasteurii*, and *Klebsiella aerogenes*, have culminated in the following model of urease activation [25], [26]. Urease initially folds in an apo-form to which a complex of three additional accessory proteins (UreD, UreF, and UreG) binds. UreG is a GTPase that forms a dimer and contains a nickel-binding site at its dimer interface. The accessory protein complex increases exposure of the apo-active site, resulting in carbamylation of the active site lysine and nickel insertion by a process that requires carbon dioxide and GTP hydrolysis. Nickel is delivered to UreG by the nickel-binding chaperone UreE, which acquires the metal from an imported pool of nickel. It is presently not clear if UreE delivers nickel to the accessory protein complex before or after it binds to apo-urease. Once the active enzyme is formed, the accessory protein complex dissociates.

The bacterial mechanism of urease maturation is predicted to occur in fungal cells. Homologues of the bacterial UreDFG proteins (Ure467) and a nickel importer (Nic1) exist in *C. neoformans* and are essential for full urease activity [22]. It appears that fungi lack a nickel chaperone with the Ure7 GTPase potentially having a dual GTPase/nickel chaperone role, as it contains a histidine-rich nickel-binding domain [22]. As with other metal chaperones, an intriguing question is where in the cell Ure7 initially binds nickel and whether this involves a direct interaction with the Nic1 nickel importer. The complexity of urease maturation therefore suggests that this pathway is a potential target for chemical intervention, in addition to the development of inhibitors of the mature enzyme, including nickel chelators and substrate analogues [27].

## Perspectives

The model of urease function during fungal infections is based on studies of two pathogenic fungi and the extrapolation of work with bacterial pathogens. However, nothing is known about the role of this enzyme during infection by other urease-positive fungi that infect the lungs, such as *Aspergillus fumigatus*, *Histoplasma capsulatum*, *Blastomyces dermatitidis*, and *Paracoccidioides brasiliensis* [28], [29]. It would also be interesting to determine why some but not all dermatophyte species that cause skin infections are urease positive [30]. Future work needs to address whether and to what extent urease is required for the virulence of these organisms, and if it is required, whether this simply relates to the provision of a nitrogen source for the pathogen or involves more complex pH-mediated damage to host cells and immunity. Potentially, such studies will enable similar strategies to be developed to treat a wide range of disparate bacterial and fungal infections.
